# *Ginkgo biloba* extract protects early brain injury after subarachnoid hemorrhage via inhibiting thioredoxin interacting protein/NLRP3 signaling pathway 

**DOI:** 10.22038/ijbms.2020.42834.10090

**Published:** 2020-10

**Authors:** Chuan Du, Chao Xi, Chunxiao Wu, Jichang Sha, Jinan Zhang, Chao Li

**Affiliations:** 1 Neurosurgery Department, Zhangqiu District People’s Hospital, Jinan 250200, China; 2 Cardiothoracic Surgery Department, Zhangqiu District People’s Hospital, Jinan250200, China; 3 Pharmacy Intravenous Admixture Services, Zhangqiu District People’s Hospital, Jinan 250200, China; 4 ENT Department, Zhangqiu District People’s Hospital, Jinan 250200, China; 5 Neurosurgery Department, Qilu Hospital of Shandong University, Jinan 250012, China

**Keywords:** Brain injury, Ginkgo biloba extract, Inflammation, Oxidative stress, Subarachnoid hemorrhage

## Abstract

**Objective(s):**

To investigate the effect of *Ginkgo biloba *extract EGb761 in early brain injury (EBI) after subarachnoid hemorrhage (SAH) and its mechanism.

**Materials and Methods:**

The SAH rat model was constructed and pre-treated with EGb761.The neurological function, severity of SAH, water content of brain tissue, damage degree of the blood-brain barrier, related indexes of oxidative stress, and the level of inflammatory cytokines were compared among the groups. The expression of TXNIP/NLRP3 signaling pathway-related proteins in brain tissues was detected by Western blot.

**Results:**

After SAH modeling, the neurological function score was significantly reduced, the degree of brain injury, levels of oxidative stress, inflammatory factors, expression of NLRP3 and TXNIP were all increased. Compared with the SAH rats, the neurological function score of rats pre-treated by EGb761 was higher, the degree of brain injury, levels of oxidative stress and inflammatory factors, expression of NLRP3 and TXNIP were all lower.

**Conclusion:**

EGb761 could protect neurological injury after SAH and its mechanism may be that EGb761 could inhibit the activation of the TXNIP/NLRP3 signaling pathway and inflammatory reaction after oxidative stress.

## Introduction

Subarachnoid hemorrhage(SAH) is a type of cerebral hemorrhage with an incidence of about 5% of strokes, with high morbidity and mortality (1). Early brain injury (EBI) is a major factor affecting the prognosis and survival of SAH. EBI refers to a series of pathological changes such as increased intracranial pressure, destruction of the blood-brain barrier, autophagy, apoptosis, inflammatory reaction, and oxidative stress in the early stage (72 hr) after cerebral hemorrhage (2). The degree of EBI is closely related to the prognosis of neurological function (3). Therefore, effectively protecting EBI is of great significance for improving the prognosis of patients with SAH. 

*Ginkgo biloba* extract (EGb 761) is a natural product from *G. biloba* that has been reported to have a variety of pharmacological effects including scavenging free radicals and antioxygenation (4, 5). EGb761 has been widely used in the treatment of various cardiovascular and cerebrovascular diseases especially myocardial ischemia-reperfusion and cerebral ischemia-reperfusion injury (6, 7), however, there are few studies on EGb761 protection of brain injury after SAH and its molecular mechanism. Additionally, thioredoxin interacting protein (TXNIP)/NOD like receptors pyrin domain-containing 3(NLRP3) signal pathway has been considered a classical pathway of inflammation and plays a key role in oxidative stress injury. In this study, the authors intended to investigate the effect of EGb761 on EBI in the SAH rat model and the possible mechanism related to the TXNIP/NLRP3 pathway.

## Materials and Methods

Experimental animals

Ninety Sprague Dawley (SD) rats were provided by Shanghai Slack Laboratory Animal Co., Ltd., male, weighing 280–320 g, 8–10 weeks old. The rats were housed in a single breeding cage at an ambient temperature of 21 to 24 ^°^C and a humidity of 40 to 50%, free to drink and eat.

Animal grouping and intervention

Ninety SD rats were randomly divided into the control group, sham group, SAH group, EGb761 low dose group (20 mg/kg group), and EGb761 high dose group (40 mg/kg group), 18 rats in each group. The rats in EGb761 low dose group and EGb761 high dose group were given 20 mg/kg and 40 mg/kg EGb761(Dr Willmar Schwabe GmbH&Co.KG, Germany), respectively by gavage while the control group, sham group and SAH group were given the same volume of saline by gavage, once a day for 14 days. 2 hr after the last gavage, the rats in SAH group, EGb761 low dose group, and EGb761 high dose group were conducted for SAH modeling.

***SAH modeling***

***The SAH model was conducted by internal carotid artery puncture ***(8). Rats were anesthetized by intraperitoneal injection of 1% sodium pentobarbital (50 mg/kg). The head and limbs were fixed on the back and the front of the neck was depilated. The median of the neck was incised to expose the right common carotid artery (CCA), external carotid artery (ECA), and internal carotid artery (ICA). Ligation cuts the ECA and the anastomosis between ECA and ICA. A 3-0 single-strand nylon thread was placed from the carotid artery into the intracranial segment of the internal carotid artery, causing subarachnoid hemorrhage. After 15 sec, the nylon thread was pulled out, the carotid artery stump was ligated to restore blood flow, and the neck incision was sutured. In rats of the sham group only the median of the neck was incised to expose the carotid artery.

Neurological function score

24 hr after SAH, neurobehavioral scores were performed according to the scoring rules of Sugawara *et al. *(9), including: autonomous exercise, symmetry of limb movement, forepaw extension, climbing ability, limb proprioception, and tentacle response. The lower the score, the more severe the loss of neurological function.

SAH severity score and HE staining

After completion of neurological function assessment, 6 rats were randomly selected from each group, and sacrificed by decapitation after deep anesthesia. The brain tissue was obtained on ice, and the SAH severity was evaluated (9); the brain was divided into six parts: the left and right forehead, the left and right abdomen, and the upper and lower brain stem. According to the blood volume of the subarachnoid space, each part was scored between 0 to 3 points and the sum taken as the SAH severity score. Part of the brain tissue was made into paraffin sections (4 μm) after paraffin embedding, then dewaxed by xylene, hydrated by gradient ethanol, and stained by HE. The pathological characteristic was observed under an electron microscope.

Water content of brain tissue

The brain tissue after being used for the SAH severity score was weighed by an electronic balance for the wet weight (W), then the dry weight (D) was weighed after being placed in a 100 ^°^C incubator for 72 hr: water content = (W - D) /W × 100%.

Brain tissue blood-brain barrier destruction test

After completion of neurological function assessment, 2% Evans Blue (EB, China Biyuntian Biotechnology Co., Ltd.), 5 ml/kg was injected into the femoral vein of the rats in each group. After 1 hr, the rats were sacrificed by decapitation, and the brain tissue of the temporal lobe was isolated and weighed. After homogenization in 3 ml PBS, and centrifuge at 15000 g for 30 min, an equal volume of trichloroacetic acid-ethanol (1:3) mixture was added into the supernatant, incubated at 4 ^°^C overnight. The absorbance (OD) was measured at a wavelength of 620 nm. Brain tissue EB content (μg/g) was calculated from the standard curve.

Serum inflammatory factor test

After completion of the neurological function assessment, 1 ml of blood was taken from the tail vein of the rats. The levels of IL-1β and IL-18 in serum were determined by enzyme-linked immunosorbent assay (ELISA) kit (Shanghai Huzhen Industrial Co., Ltd., China). The operation was carried out in accordance with the manufacturer’s instructions.

Oxidative stress indexes detection

After completion of the neurological function assessment, the brain tissue at the bottom of the temporal lobe was isolated after deep anesthesia and decapitation in each group. The brain tissue was lysed, homogenized, centrifuged, and quantified. According to the instructions, MDA content in brain tissue was detected by the thiobarbituric acid method, GPx activity was detected by chemical colorimetry, and SOD activity was determined by xanthine oxidase method.

Immunoprecipitation and Western blot

Part of the brain tissue at the bottom of the temporal lobe was used for detection of protein expression. Brain tissue samples were weighed and homogenated by PMSF, and the total protein was extracted using the PIRA solution. Part protein was used to do co-immunoprecipitation (Co-IP), while the other was used to do Western blot analysis. After incubation with anti-TXNIP antibody (1:200) at 4 ^°^C overnight, pretreated protein A/G agarose was added at 4 ^°^C overnight. After being washed by IP buffer and centrifugation at 12000 g for 5 min at 4 ^°^C, the supernatant was taken, and 5 × sodium dodecyl sulfate (SDS) loading buffer was added for elution. After separation with an SDS gel, it was transferred to a polyvinylidene fluoride (PVDF) membrane for Western blot analysis.

Western blot was also used to detect the relative expression of protein. 40 μg protein samples were separated by SDS-PAGE. After transfection into a PVDF membrane, the membrane was blocked in TBST buffer containing 5% skimmed milk for 1 hr at room temperature. Primary antibodies were incubated at 4 ^°^C overnight. And the secondary antibodies were incubated at room temperature for 1 hr. ECL detection reagent was used to observe protein bands and the relative expression of NLRP3, ASC, caspase-1 p10, and TXNIP proteins (β-actin was considered as internal reference) were calculated using Image J software.

Statistical analysis

Statistical analysis was performed by SPSS 19.0 software. Measurement data conforming to the normal distribution were expressed as mean±standard deviation, independent samples *t*-test was used for comparing the average of two groups, and one-way analysis of variance was performed among multiple groups; If *P*<0.05 then the difference was considered statistically significant.

## Results

HE staining and SAH severity score after SAH

**Figure 1:**
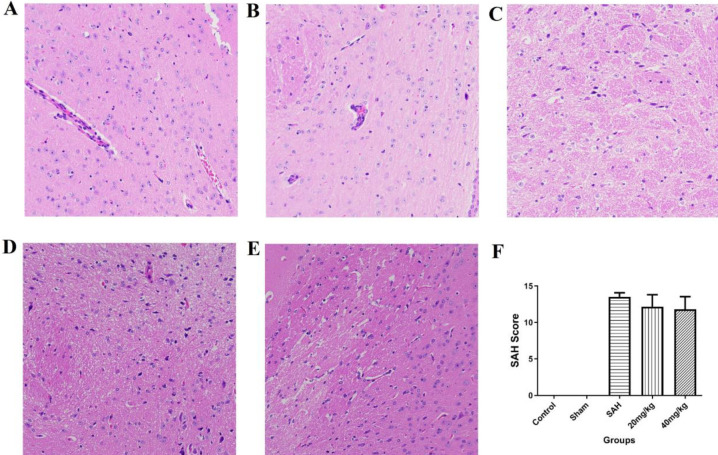
HE staining of brain tissue and SAH score of each group. A: Control group (200×); B: Sham group (200×); C: SAH group (200×); D: 20 mg/kg EGb761 group (200×); E: 40 mg/kg EGb761 group (200×); F: SAH severity score. *P<0.05 vs control group, #*P*<0.05 vs Sham group, $*P*<0.05 vs SAH group

SAH: subarachnoid hemorrhage; EGb: *Ginkgo biloba* extract; HE: Hematein Eosin

HE staining results were shown in Figure 1. Compared to the control and sham group, the brain tissue in the SAH group was disordered and the cells were loosely arranged with a wrinkled nucleus which was close to apoptosis. The brain tissue structure was improved in the 20 mg/kg group and some cells had wrinkled nucleus, while the brain tissue structure in 40 mg/kg group was significantly improved and most cells had normal structure. The amount of SAH bleeding in rats was quantified to assess the severity of SAH (Figure 1F). There was no significant bleeding or blood clots observed in the control and sham groups, so the scores were all 0. In the SAH, 20 mg/kg and 40 mg/kg groups, diffuse hemorrhages and blood clots were observed in the brain tissue of the skull, and the SAH scores were significantly higher than control and sham groups, but the differences among SAH, 20 mg/kg and 40 mg/kg groups were not statistically significance (*P*>0.05).

***Neurological function score, brain water content, and blood-brain barrier destruction in each group after SAH***

**Figure 2:**
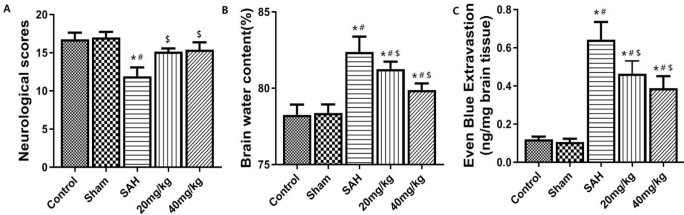
Degree of brain injury of each group of of neurological function,brain water content and blood brain barrier. A: Neurological function score. B: Brain tissue water content. C: Evans blue exudation. *P<0.05 vs control group, #*P*<0.05 vs Sham group, $*P*<0.05 vs SAH group

The results of neurological function scores, brain water content, and blood-brain barrier destruction are shown in Figure 2. SAH group showed significant neurological impairment compared with the Sham group and control (*P*<0.05). Compared with the SAH group, the neurological impairment of 20 mg/kg and 40 mg/kg groups were significantly improved (*P*<0.05, Figure 2A). Compared with the Sham group, the brain water content and EB exudation in the SAH group were significantly increased (*P*<0.05, Figures 2B and 2C). Compared with the SAH group, the brain water content and EB exudation of 20 mg/kg and 40 mg/kg groups were significantly lower (*P*<0.05).

Change of NLRP3 inflammatory body pathway in brain tissue after SAH

**Figure 3:**
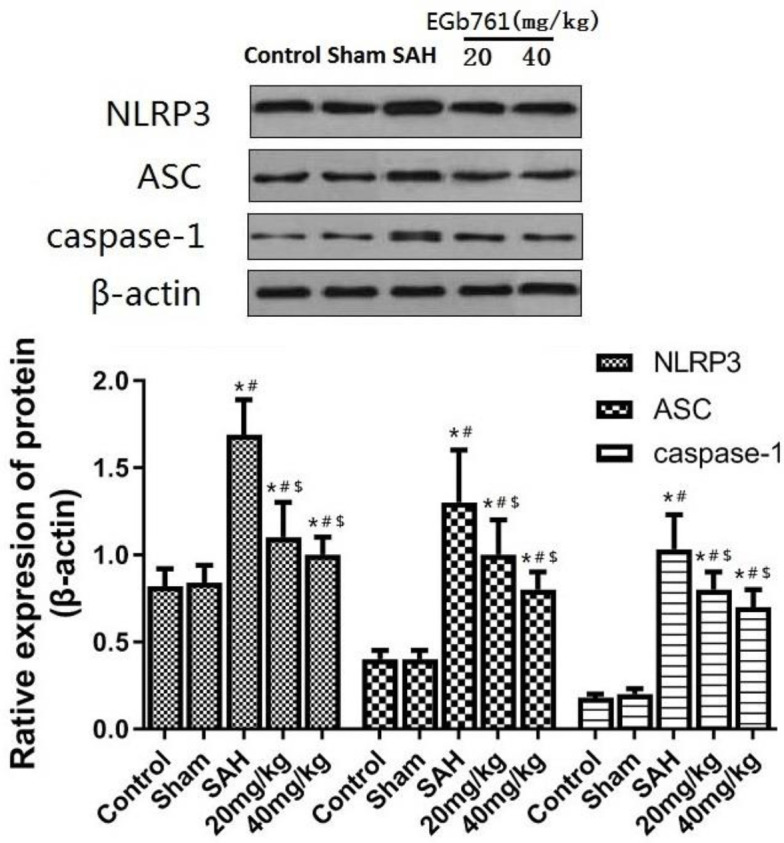
Expression of NLRP3 inflammatory body pathway-associated proteins in brain tissue of NLRP3,ASC and caspase-1. *P<0.05 vs control group, #*P*<0.05 vs Sham group, $*P*<0.05 vs SAH group

NLRP3: NOD like receptors pyrin domain-containing 3; SAH: subarachnoid hemorrhage

Western blot analysis of brain tissue NLRP3 inflammatory corpuscle pathway-associated protein expression (Figure 3): Compared with control and sham group, the relative protein expression of NLRP3, ASC and caspase-1 in SAH group was significantly higher (*P*<0.05), which showed the activation of NLRP3 inflammasome after SAH. Compared with the SAH group, the relative expressions of NLRP3, ASC, and caspase-1 in EGb761 20 mg/kg and 40 mg/kg groups were significantly lower (*P*<0.05).

Change of inflammatory cytokine level after SAH

**Figure 4:**
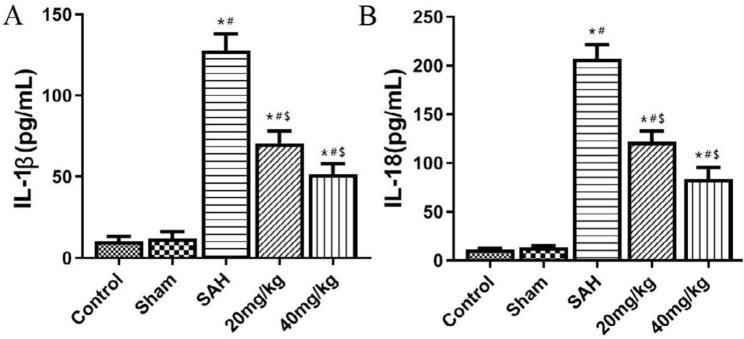
NLRP3 inflammatory body-dependent cytokine levels in serum ofIL-1β and IL-18. A: Serum IL-1β content; B: Serum IL-18 level. *P<0.05 vs control group, #*P*<0.05 vs Sham group, $*P*<0.05 vs SAH group

NLRP3: NOD like receptors pyrin domain-containing 3; SAH: subarachnoid hemorrhage

The serum inflammatory cytokines were detected by ELISA. The results in Figure 4 show that serum IL-1β and IL-18 levels are significantly increased in the SAH group compared with control and sham groups (*P*<0.05). Compared with the SAH group, serum IL-1β and IL-18 levels in 20 mg/kg and 40 mg/kg groups were significantly lower (*P*<0.05).

***Changes in oxidative stress status of brain tissue after SAH***

**Figure 5:**
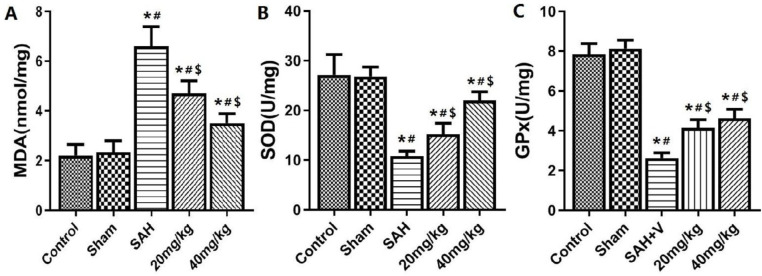
Brain tissue oxidative stress indicators of MDA,SOD and GPx. A: MDA content; B: GPx content. C: SOD content. *P<0.05 vs control group, #*P*<0.05 vs Sham group, $*P*<0.05 vs SAH group

The quantitative analysis results of oxidative stress indexes are shown in Figure 5: Compared with the control and sham group, the MDA content in the brain tissue of the SAH group was significantly increased, and the activities of the antioxidant enzymes GPx and SOD were significantly decreased (*P*<0.05). Compared with the SAH group, MDA was significantly lower and GPx and SOD were significantly higher in 20 mg/kg and 40 mg/kg groups(*P*<0.05).

***Changes in TXNIP/NLRP3 signaling pathway in brain tissue after SAH***

**Figure 6:**
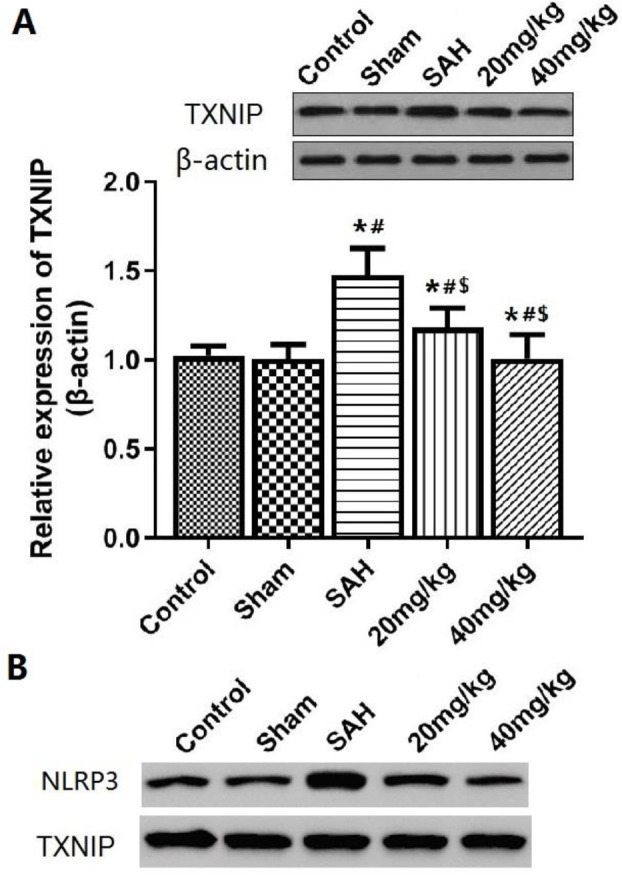
TXNIP expression and TXNIP-NLRP3 interaction in brain tissue after SAH

***The results of Western blot shown in*** Figure 6 ***suggest that compared with control and sham groups, the expression of TXNIP protein in the brain tissue of the SAH group was significantly increased*** *(P*<0.05). EGb721 20 mg/kg and 40 mg/kg treatments significantly inhibited the expression of TXNIP(*P*<0.05). The results of co-immunoprecipitation also confirmed (Figure 6B) that NLRP3 interacted with TXNIP in brain tissue of SAH rats, and the interaction between NLRP3 and TXNIP was inhibited by EGb761.

SAH: subarachnoid hemorrhage

MDA: malondialdehyde; GPx:glutathion peroxidase; SOD:superoxide dismutase

A: Expression of TXNIP in brain tissue analyzed by Western Blot; B: Immunoprecipitation analysis of the interaction of TXNIP and NLRP3 in brain tissue. *P<0.05 vs control group, #*P*<0.05 vs Sham group, $*P*<0.05 vs SAH group

TXNIP: thioredoxin interacting protein; NLRP3: NOD like receptors pyrin domain-containing 3; SAH: subarachnoid hemorrhage

## Discussion

A lot of research shows that EGb761 could be used for treatment of ischemia injury in lung, brain, and kidney, *etc. *(10), and it also has a neuroprotective effect in some kinds of brain diseases(11, 12). Only a few researchers reported the protective effect of EGb761 on the brain after SAH: Sun *et al*. (13) study shows that *G. biloba* extract could relieve cerebral vasospasm and cerebral ischemic injury after SAH by reversing the pathological alteration of nitric oxide. Yu *et al.* (14) study shows that EGb761 could ameliorate EBI after SAH and inhibit the neuronal apoptosis by inhibiting the activation of the Akt pathway. The degree of EBI was mainly reflected by the degree of neurological deficit, cerebral edema, damage of the blood-brain barrier, and neuroinflammatory response (15). In this study, the brain edema and blood-brain barrier destruction occurred in the rats 24 hr after SAH, and the neurological function score was also significantly reduced, confirming the occurrence of EBI in the acute phase after SAH. The treatment of EGb761 can significantly improve cerebral edema, blood-brain barrier, and brain tissue lesions, suggesting that EGb761 has some neuroprotective effects for EBI after SAH, which is similar to the existing research.

NLRP3, an innate immune receptor, belongs to the family of NOD-like receptors (NLRs) and plays an important role in the development of inflammatory responses. The activation of NLRP3 can recruit ASC and pro-caspase-1 to form NLRP3 inflammasome. Activation and release of caspase-1 to the extracellular region promotes maturation and release of IL-1β and IL-18 (16). Study has found that NLRP3 inflammasome is involved in aggravating the neuroinflammatory response of SAH and spontaneous intracranial hemorrhage, and its activation is mainly located in microglia (17, 18), while microglia and astrocytes play a key role in the release of pro-inflammatory factors and aggravation of secondary brain injury (19). Previous studies have also confirmed that inhibition of NLRP3 inflammasome activation in the acute phase after SAH has a strong neuroprotective effect which was related to the reduction of inflammatory cytokines (20). In this study, it was shown that the expression of NLRP3 in brain tissue was significantly up-regulated and the serum IL-1β and IL-18 levels were significantly increased 24 hr after SAH, suggesting that NLRP3 inflammasome activation after SAH may participate in EBI by regulating neuroinflammatory reaction; and the treatment of EGb761 can significantly reduce the expression of NLRP3 and inflammatory cytokines levels, suggesting that EGb761 may play a neuroprotective effect by inhibiting activation of NLRP3 inflammasome.

TXNIP, a member of the thioredoxin system that regulates oxidative stress in the body, modulates oxidative stress by inhibiting thioredoxin (TRX) under oxidative stress and stimulates inflammation and apoptosis by interacting with excess ROS(21). TXNIP is necessary for NLRP3 inflammasome activation, and NLRP3 expression is elevated when TXNIP is up-regulated(22). Zhou *et al. *(23) confirmed the targeted regulatory relationship between TXNIP and NLRP3: ROS can induce TXNIP to dissociate from TRX and bind to NLRP3 to form TXNIP-NLRP3, which interacts with each other, leading to activation of NLRP3 inflammasome(24). Therefore, the mechanism of TXNIP and NLRP3 involved in brain injury after SAH is that ischemia and hypoxia caused by SAH leads to an imbalance of oxidative-reduction system, resulting in the excessive binding of TXNIP to NLRP3, thereby activating NLRP3 inflammasome and further opening its downstream signaling pathway, promoting local tissue inflammation and aggravating brain tissue damage. In this study, it was shown that oxidative stress indexes and expression of TXNIP increased significantly after SAH, and treatment by EGb761 could inhibit oxidative stress and expression of TXNIP, then inhibited the interaction of TXNIP with NLRP3 and then NLRP3 inflammasome activation. Therefore, it could be speculated that EGb761 may play a neuroprotective effect by inhibiting the interaction of TXNIP with NLRP3 and the activation of NLRP3 inflammasome.

## Conclusion

The results of the present study showed that EGb761 could inhibit the oxidative stress injury and act as a neuroprotective after SAH, the mechanism may be that EGb761 could reduce the expression of inflammatory corpuscle-related proteins TXNIP and NLRP3 and inhibit the activation of inflammasome signal pathway after their interaction.

## References

[1] Lucke-Wold BP, Logsdon AF, Manoranjan B, Turner RC, McConnell E, Vates GE et al (2016). Aneurysmal subarachnoid hemorrhage and neuroinflammation: A comprehensive review. Int J Mol Sci.

[2] Cahill J, Zhang JH (2009). Subarachnoid hemorrhage: is it time for a new direction?. Stroke.

[3] Fujii M, Yan J, Rolland WB, Soejima Y, Caner B, Zhang JH (2013). Early brain injury, an evolving frontier in subarachnoid hemorrhage research. Transl Stroke Res.

[4] Tian J, Liu Y, Chen K (2017). Ginkgo biloba Extract in vascular protection: molecular mechanisms and clinical applications. Curr Vasc Pharmacol.

[5] Savaskan E, Mueller H, Hoerr R, von Gunten A, Gauthier S (2018). Treatment effects of Ginkgo biloba extract EGb 761(R) on the spectrum of behavioral and psychological symptoms of dementia: meta-analysis of randomized controlled trials. Int Psychogeriatr.

[6] Li T, Zhang Y, Tian J, Yang L, Wang J (2019). Ginkgo biloba pretreatment attenuates myocardial ischemia-reperfusion injury via mitoBKCa. Am J Chin Med.

[7] Erbil G, Ozbal S, Sonmez U, Pekcetin C, Tugyan K, Bagriyanik A et al (2008). Neuroprotective effects of selenium and Ginkgo biloba extract (EGb761) against ischemia and reperfusion injury in rat brain. Neurosciences (Riyadh).

[8] Bederson JB, Germano IM, Guarino L (1995). Cortical blood flow and cerebral perfusion pressure in a new noncraniotomy model of subarachnoid hemorrhage in the rat. Stroke.

[9] Sugawara T, Ayer R, Jadhav V, Zhang JH (2008). A new grading system evaluating bleeding scale in filament perforation subarachnoid hemorrhage rat model. J Neurosci Methods.

[10] Li Y, Xiong Y, Zhang H, Li J, Wang D, Chen W (2017). Ginkgo biloba extract EGb761 attenuates brain death-induced renal injury by inhibiting pro-inflammatory cytokines and the SAPK and JAK-STAT signalings. Sci Rep.

[11] Singh SK, Srivastav S, Castellani RJ, Plascencia-Villa G, Perry G (2019). Neuroprotective and antioxidant effect of Ginkgo biloba extract against AD and other neurological disorders. Neurotherapeutics.

[12] Guan ZF, Zhang XM, Tao YH, Zhang Y, Huang YY, Chen G (2018). EGb761 improves the cognitive function of elderly db/db(-/-) diabetic mice by regulating the beclin-1 and NF-kappaB signaling pathways. Metab Brain Dis.

[13] Sun BL, Xia ZL, Yang MF, Qiu PM (2000). Effects of Ginkgo biloba extract on somatosensory evoked potential, nitric oxide levels in serum and brain tissue in rats with cerebral vasospasm after subarachnoid hemorrhage. Clin Hemorheol Microcirc.

[14] Yu T, Fan Y, Xu Y, Xu L, Xu G, Cao F (2018). Standardized Ginkgo biloba extract EGb 761(R) attenuates early brain injury following subarachnoid hemorrhage via suppressing neuronal apoptosis through the activation of Akt signaling. Biomed Pharmacother.

[15] Topkoru B, Egemen E, Solaroglu I, Zhang JH (2017). Early brain injury or vasospasm? An overview of common Mechanisms. Curr Drug Targets.

[16] Xie Q, Shen WW, Zhong J, Huang C, Zhang L, Li J (2014). Lipopolysaccharide/adenosine triphosphate induces IL1beta and IL-18 secretion through the NLRP3 inflammasome in RAW2647 murine macrophage cells. Int J Mol Med.

[17] Liu HD, Li W, Chen ZR, Hu YC, Zhang DD, Shen W (2013). Expression of the NLRP3 inflammasome in cerebral cortex after traumatic brain injury in a rat model. Neuro Chem Res.

[18] Pan Y, Chen XY, Zhang QY, Kong LD (2014). Microglial NLRP3 inflammasome activation mediates IL-1beta-related inflammation in prefrontal cortex of depressive rats. B rain Behav Immun.

[19] Farina C, Aloisi F, Meinl E (2007). Astrocytes are active players in cerebral innate immunity. Trends Immunol.

[20] Chen S, Ma Q, Krafft PR, Hu Q, Rolland WN, Sherchan P (2013). P2X7R/cryopyrin inflammasome axis inhibition reduces neuroinflammation after SAH. Neurobiol Dis.

[21] Tschopp J, Schroder K (2010). NLRP3 inflammasome activation: The convergence of multiple signalling pathways on ROS production?. Nat Rev Immunol.

[22] Iyer SS, He Q, Janczy JR, Elliott EI, Zhong Z, Olivier AK (2013). Mitochondrial cardiolipin is required for Nlrp3 inflammasome activation. Immunity.

[23] Zhou R, Yazdi AS, Menu P, Tschopp J (2011). A role for mitochondria in NLRP3 inflammasome activation. Nature.

[24] Gao P, Meng XF, Su H, He FF, Chen S, Tang H (2014). Thioredoxin-interacting protein mediates NALP3 inflammasome activation in podocytes during diabetic nephropathy. Biochim Biophys Acta.

